# InnerEye-HS: a disease-agnostic clinical tool for hippocampal segmentation

**DOI:** 10.1093/braincomms/fcag183

**Published:** 2026-05-21

**Authors:** Anna Schroder, James Moggridge, Hamza A Salhab, Caroline Micallef, Jiaming Wu, Melissa Bristow, Fernando Pérez-García, Javier Alvarez-Valle, Sjoerd B Vos, Tarek A Yousry, John S Thornton, Frederik Barkhof, John S Duncan, Daniel C Alexander, Matthew Grech-Sollars

**Affiliations:** Department of Computer Science, University College London, London WC1V 6LJ, UK; Lysholm Department of Neuroradiology, National Hospital for Neurology and Neurosurgery, University College London Hospitals NHS Foundation Trust, London WC1N 3AR, UK; Department of Translational Neuroscience and Stroke, UCL Institute of Neurology, University College London, London WC1N 3AR, UK; Lysholm Department of Neuroradiology, National Hospital for Neurology and Neurosurgery, University College London Hospitals NHS Foundation Trust, London WC1N 3AR, UK; Lysholm Department of Neuroradiology, National Hospital for Neurology and Neurosurgery, University College London Hospitals NHS Foundation Trust, London WC1N 3AR, UK; Department of Medical Physics & Biomedical Engineering, University College London, London WC1E 6BT, UK; Health Futures, Microsoft Research Cambridge, Cambridge CB1 2FB, UK; Health Futures, Microsoft Research Cambridge, Cambridge CB1 2FB, UK; Health Futures, Microsoft Research Cambridge, Cambridge CB1 2FB, UK; Western Australia National Imaging Facility, The University of Western Australia, Perth WA 6009, Australia; Lysholm Department of Neuroradiology, National Hospital for Neurology and Neurosurgery, University College London Hospitals NHS Foundation Trust, London WC1N 3AR, UK; Department of Translational Neuroscience and Stroke, UCL Institute of Neurology, University College London, London WC1N 3AR, UK; Lysholm Department of Neuroradiology, National Hospital for Neurology and Neurosurgery, University College London Hospitals NHS Foundation Trust, London WC1N 3AR, UK; Department of Translational Neuroscience and Stroke, UCL Institute of Neurology, University College London, London WC1N 3AR, UK; Department of Translational Neuroscience and Stroke, UCL Institute of Neurology, University College London, London WC1N 3AR, UK; Department of Medical Physics & Biomedical Engineering, University College London, London WC1E 6BT, UK; Department of Radiology and Nuclear Medicine, Amsterdam Neuroscience, Vrije Universiteit Amsterdam, Amsterdam UMC, Amsterdam 1105AZ, The Netherlands; Department of Epilepsy, UCL Queen Square Institute of Neurology, University College London, London WC1N 3AR, UK; Department of Computer Science, University College London, London WC1V 6LJ, UK; Department of Computer Science, University College London, London WC1V 6LJ, UK; Lysholm Department of Neuroradiology, National Hospital for Neurology and Neurosurgery, University College London Hospitals NHS Foundation Trust, London WC1N 3AR, UK

**Keywords:** Alzheimer’s disease, epilepsy, hippocampal segmentation, magnetic resonance imaging, deep learning

## Abstract

The hippocampus is subject to atrophy in both Alzheimer’s disease and temporal lobe epilepsy. Hippocampal volumes thus provide an early biomarker for these diseases. However, automated segmentation models typically lack robustness to disease-related changes in the hippocampus.

In this work, we present the InnerEye hippocampal segmentation tool (InnerEye-HS). This deep learning tool was trained on MRI scans across the Alzheimer’s disease spectrum, providing exposure to varying hippocampal size and topology. We validate the model against manually segmented hippocampi on both clinical dementia and epilepsy datasets collected in clinical settings and compare our model’s performance to four other freely available tools (Automatic Segmentation of Hippocampal Subfields (ASHS), FreeSurfer, FastSurfer and HIPPOSEG).

When compared to other freely available tools, the InnerEye-HS model provides the best Dice scores in our hospital dementia dataset (mean = 0.85 ± 0.02, *P* ≤ 0.0125), and InnerEye-HS and ASHS provided the best Dice scores in our epilepsy dataset (InnerEye-HS mean = 0.85 ± 0.02, ASHS mean = 0.84 ± 0.03). Furthermore, we found a high correlation (R^2^ = 0.85) between hippocampal volumes extracted from ground-truth segmentations and those extracted from InnerEye-HS segmentations, demonstrating the model’s ability to robustly segment the hippocampus throughout the disease time course.

In summary, we present the InnerEye-HS model and demonstrate its advantage over currently available tools. These advantages highlight the clinical utility of our tool.

## Teaser

Segmentation of the hippocampus, a small brain region, is critical for monitoring neurodegenerative diseases, e.g. Alzheimer’s disease and epilepsy. Schroder *et al*. present InnerEye-HS, a deep learning segmentation tool that delineates the hippocampus from MRI with greater accuracy and time efficiency than currently available tools, leading to direct patient benefits.

## Graphical Abstract

**Graphical Abstract:**
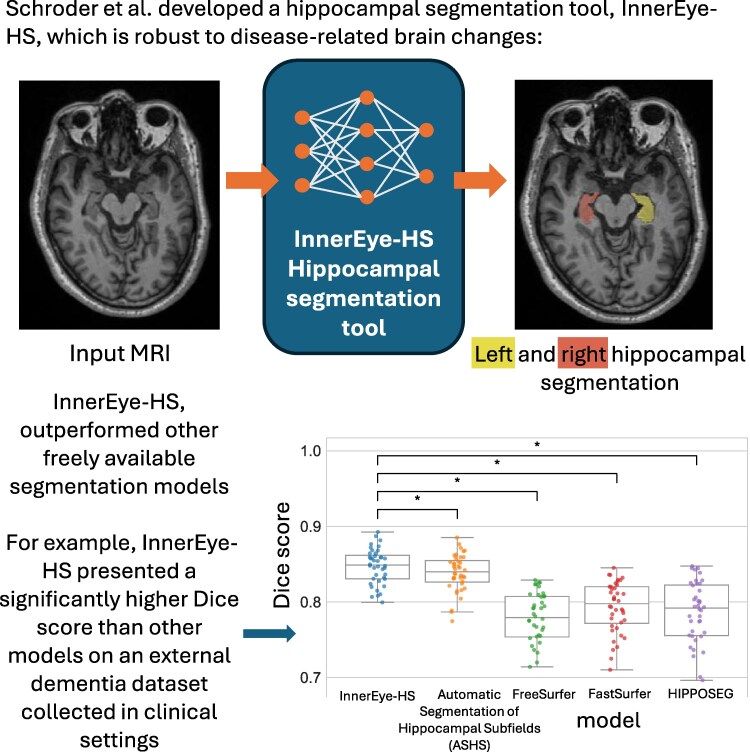

## Introduction

The hippocampus is a small, deep grey matter region whichplays a crucial role in long and short-term memory formation and retrieval.^1^ The region is composed of several subfields, each with distinct functions and vulnerability to disease.^2,3^ The shape and volume of the hippocampus are affected by neurodegenerative diseases such as Alzheimer’s Disease (Ad), where hippocampal volume provides an early biomarker of disease,^4^ and temporal lobe epilepsy with hippocampal sclerosis.^5^ Continued assessment of the hippocampus throughout the disease time course enables monitoring of progression and assessment of the impact of interventions in Ad, e.g. in clinical trials,^6^ and informs treatment options in epilepsy.^7^

Magnetic Resonance Imaging (MRI) enables inspection of the brain *in vivo* and non-invasively, enabling continued assessment of the hippocampus throughout the disease time course. Delineating the boundaries of the hippocampus from these scans enables assessment of hippocampal volume and topology. While manual segmentation remains the gold standard,^8^ it is time-consuming and costly, so there remains a clinical need for fast, accurate and robust automated segmentation tools. Automated segmentation of the hippocampus is particularly challenging due to the small and complex shape of the region, which has significant variability. This variability is exacerbated by the fact that we often want to segment the region into Ad cohorts with extensive atrophy. As a result, the hippocampus is a key region where openly available tools lack robustness, particularly in patients with conditions that affect hippocampal size and shape.

Various automated brain segmentation tools have emerged over recent years.^9,10^ Common approaches largely fall into two categories: multi-atlas segmentation and machine learning models. Multi-atlas segmentation^11-14^ registers multiple atlas images to a new image, producing a transformation field for each atlas image. This transformation field is then applied to the manually segmented image for each atlas, producing multiple segmentations for the new image. The segmentations from multiple atlases are combined. Machine learning approaches, specifically deep learning models,^15-18^ typically utilize convolutional neural networks (CNNs) to classify each image voxel, producing a segmentation mask. These models are computationally expensive to train; however, segmentation inference of new images is substantially faster than multi-atlas approaches. Hippocampal-specific segmentation tools use both multi-atlas approaches^19,20^ and deep-learning approaches.^21,22^ Other approaches also aim to further segment the hippocampus into its sub-fields.^23-25^

Despite these advances, automated segmentation tools typically fail when applied to uncurated hospital data and when they try to capture disease-related changes. This is a particular problem in the hippocampus, as it is a small, complex structure with substantial variability. Template-based models struggle to cope with disease-related variability and have long run-times, limiting their use clinically and at large scale. Deep learning models have typically been trained on healthy subjects,^26,27^ limiting their robustness to accurately segment the hippocampus in subjects with disease-related variability.

In this paper, we present our InnerEye hippocampal segmentation tool (InnerEye-HS), a deep learning tool for hippocampal segmentation which is robust to disease-related changes. We outline model training for InnerEye-HS, an ensemble of 3D-UNets, on publicly available data from an AD cohort, and validation of our model on four clinical datasets with manual hippocampal segmentations. We demonstrate InnerEye-HS’s ability to robustly segment the hippocampus across different diseases and disease stages, in both dementia and epilepsy datasets collected in clinical settings. We compare the InnerEye-HS model to four freely available segmentation tools to determine the most appropriate model for clinical implementation. We packaged the model and pre-processing steps into a Docker image, which is freely available (https://xip.uclb.com/product/innereye-hs).

## Materials and methods

### Data

Four datasets were used in this work. Research data from the Alzheimer’s Disease Neuroimaging Initiative (ADNI) were used for pre-training, fine-tuning and internal validation. Three clinical datasets were collected for external validation on hospital data: two from the National Hospital for Neurology and Neurosurgery (NHNN), and one from the Chalfont Centre for Epilepsy.

#### ADNI

We collected 1415 T1 MPRAGE MRI scans from the ADNI database (adni.loni.usc.edu). For up-to-date information, see www.adni-info.org. Data from the ADNI included three subsets: ADNI A, which was used for pre-training; ADNI B, used for fine-tuning; and ADNI C, used for internal validation. Each of these subsets had different ground truth segmentation protocols. Subject demographics for each subset are provided in Table 1. Note that ADNI A contained multiple scans per subject, one time point was selected at random for inclusion in the summary of demographics.

**Table 1:** A summary of the datasets used in this paper

Dataset	Number of scans	Segmentation technique	Diagnosis			Mean age (SD)	Female (%)	Mean MMSE (SD)
			Dementia (%)	MCI (%)	CN (%)			
ADNI A (pre-training)	1250	semi-automatic	24.6	46.4	29.0	75.9 (6.7)	43.5	26.3 (3.5)
ADNI B (fine-tuning)	135	manual	33.3	26.7	39.3	74.9 (7.8)	48.1	25.7 (4.0)
ADNI C (internal validation)	30	manual	33.3	33.3	33.3	76.8 (7.2)	30	25.4 (3.1)
NHNN dementia	20	manual	-	-	-	69.0 (14.8)	30	-
NHNN epilepsy	8	manual	Temporal Lobe Epilepsy			46.8 (24.9)	62.5	-
Chalfont	20	manual	Temporal Lobe Epilepsy			-	-	-

We report clinical diagnoses (dementia, mild cognitive impairment (MCI) and cognitively normal (CN)), Mini Mental State Examination (MMSE), age and sex where possible. All demographic information is available in the ADNI dataset. MMSE was not available for either of the NHNN datasets. All NHNN dementia dataset patients had been referred to a memory clinic, but specific diagnoses were not available. All NHNN epilepsy patients had been diagnosed with temporal lobe epilepsy.

ADNI A contains semi-automatic segmentations and is the largest subset (1250 T1 scans from 386 subjects). Scans had been pre-processed by ADNI using a combination of Gradwarp, B1-field correction and N3-bias correction. Ground truth segmentations,^28^ downloaded from ADNI, had previously been created semi-automatically using the Medtronic surgical navigation technology, a high-dimensional brain-mapping tool. The semi-automatic segmentation tool, which has previously been validated by Hsu *et al*.,^29^ involves manual placement of 22 control points on landmarks on each hippocampus, followed by an automatic technique to produce the segmentation boundaries. This automatic technique involved a fluid image transformation to match individuals to template brain. Finally, all segmentations were checked by qualified reviewers to determine whether a boundary ‘failed’. Further information on the ADNI semi-automatic hippocampal segmentations is detailed in https://adni.bitbucket.io/reference/docs/UCSFSNTVOL/UCSFMRI_Analysis.pdf. This approach enabled the collection of a relatively large dataset, which we used to pre-train the InnerEye-HS segmentation model. Clinicians in our team noted that the semi-automatic segmentations deviated from the Harmonized Protocol for hippocampal segmentation. For example, it was noted that the ADNI segmentations, in some cases, under-segmented the subiculum and the hippocampus proper, as well as included portions of the choroid plexus.

ADNI B is a smaller subset (135 scans from independent subjects) and contains manual segmentations downloaded from the Harmonized Protocol (HarP) dataset,^30^ where hippocampi were segmented by five raters according to the agreed harmonized protocol,^31^ followed by an independent quality check. We fine-tuned our model with this dataset, such that the model’s segmentations are more closely aligned with the harmonized protocol. Both ADNI A and ADNI B were split into training, validation, and testing splits using an 80:10:10 ratio, respectively.

ADNI C contains 30 MRI scans manually segmented by a local clinician and confirmed by a consultant neuroradiologist. This dataset was used to internally validate the InnerEye-HS segmentation model. Images were evenly sampled from subjects with cognitively normal (CN), mild cognitive impairment (MCI) and dementia diagnoses, providing an assessment of InnerEye-HS across the disease spectrum.

The manual segmentation protocol for ADNI C was as follows: the segmented body included the hippocampus proper, alveus, and subiculum. Fimbria and choroid plexus were excluded. Anteriorly, the hippocampus was differentiated from the amygdala by the presence of the alveolar covering. The medial boundaries were defined by the hippocampal and uncal fissures. At the lateral aspect, the limits were determined by the temporal horn of the lateral ventricle and/or white matter of the temporal stem. Posteriorly, the segmentation relied on the grey-white interface to define the borders. This segmentation protocol was consistent with the harmonized protocol, with the exception of one region, the fimbria, which was included in the harmonized protocol but excluded from our manual protocol.

#### NHNN

Two datasets of 3T T1 MPRAGE scans were collected locally from the NHNN: one collected at a dementia clinic (*N* = 20); the other at an epilepsy clinic (*N* = 8). All patients in the epilepsy dataset had been diagnosed with temporal lobe epilepsy. All patients in the dementia dataset had been referred to a memory clinic. Their clinical diagnoses were not available, however, all except one of these patients were clinically seen to have disease-related atrophy. The hippocampus was manually segmented from each MRI scan by the same clinicians, using the same protocol as the ADNI C dataset.

For both datasets, scans were collected on a Siemens MAGNETOM Prisma Fit scanner (3T). The imaging protocol for the dementia dataset was: Echo time (TE) = 2.95 ms, repetition time (TR) = 2300 ms, inversion time (TI) = 900 ms, field of view (FOV) = 270 mm× 254 mm × 211 mm, voxel size = 1.1 mm × 1.1 mm × 1.2 mm, acceleration factor = 2. For one patient, a different protocol was used with voxel size 1.3 mm × 1.3 mm × 1.2 mm.

For the epilepsy dataset, the imaging protocol was: TE = 2.4 ms, TR = 1540 ms, TI = 900 ms, FOV = 240 mm × 240 mm × 160 mm, voxel size = 0.8 mm × 0.8 mm × 1.0 mm, acceleration factor = 2. Ethical approval for the research study using data from UCLH was given through the hospital Data Access Process for Research^32^ (DAPR).

#### Chalfont centre for epilepsy

A dataset of 20 3T T1 scans from unilateral refractory temporal lobe epilepsy patients was randomly sampled from the validation set described by Postma *et al*..^33^ Scans were collected on a GE Discovery MR750, using a 3D T1-weighted inversion-recovery fast spoiled gradient recalled echo protocol: TE = 3.1 ms, TR = 7.4 ms, TI = 400 ms, FOV = 224 mm × 256 mm × 256 mm, voxel size = 1 mm × 1 mm × 1 mm, parallel imaging acceleration = 2.

The hippocampus was manually segmented from these scans by the same clinicians using the same protocol as the NHNN and ADNI C datasets. Ethical approval for the research study was given through DAPR.^32^

### InnerEye hippocampal segmentation model (InnerEye-HS)

InnerEye is a toolbox for training and evaluating deep learning models on 3D medical images. It has shown success in, for example, segmentation models of structures in the head and pelvis for radiotherapy planning for head, neck and prostate cancers in CT scans.^34^

We used InnerEye to train a model for hippocampal segmentation of T1-weighted MRI. The model followed the training described by Oktay *et al*.^34^ and consists of a five-fold ensemble model of 3D U-nets. MRI scans are processed with an input patch size of (128, 176, 176), which ensures that part of the hippocampus is always included in all input patches.

The model was trained using a soft dice loss and optimized with the Adam optimizer. Our InnerEye model for hippocampal segmentation was pre-trained using the large ADNI A dataset, and we fine-tuned each ensemble model using the ADNI B dataset. The learning rate for pre-training was set to 1e−3, and the learning rate for fine-tuning was selected to maximize the Dice score on the ADNI B validation dataset (learning rate = 1e−4). We will refer to this fine-tuned model as InnerEye-HS in this paper.

We pre-processed the images to align more closely with the ADNI dataset. Images were pre-processed using FSL (v5.0.8)^35^ to reorient the images to MNI orientation, automatically crop the images, correct the bias field, and resample the images to the same resolution as the ADNI B dataset (1 mm isotropic). Following InnerEye-HS inference, post-processing steps were carried out to return the image segmentations to the original image space and resolution. A threshold of 0.5 was used to binarize the resampled segmentations.

We packaged the pre-processing, InnerEye-HS inference and post-processing steps into a Docker container to aid research uptake and clinical implementation. The Docker InnerEye-HS model, including model weights, is hosted at https://xip.uclb.com/product/innereye-hs.

### Model comparison

We compare InnerEye-HS to a selection of commonly used tools for hippocampal segmentation. This selection spans whole-brain and hippocampal-specific tools, including multi-atlas registration and deep learning approaches.

The Automatic Segmentation of Hippocampal Subfields (ASHS)^25^ (v2.0.0) is a multi-atlas label fusion technique developed for segmenting hippocampal subfields. This model requires both a T1- and T2-weighted scan as input to segment the hippocampal subfields. However, additional atlases are provided to segment the whole hippocampus from a T1-weighted scan alone. Different atlases were used depending on the condition of the patients (dementia or epilepsy) in the dataset being segmented: the Penn Memory Centre T1-Only Atlas for T1-weighted 3T MRI^36^ was used for the ADNI C and NHNN dementia datasets; the Penn Temporal Lobe Epilepsy T1-MRI Whole Hippocampus ASHS Atlas was used for the NHNN epilepsy dataset.

FreeSurfer^12^ (v7.4.1) is a multi-atlas registration model for whole brain parcellation. FastSurfer^18^ (v2.2.0) is a deep-learning model trained in the output of FreeSurfer (v6.0). We used the segmentation-only tag to reduce running times. For both FastSurfer and FreeSurfer, default settings were used in brain parcellation.

The HIPPOSEG^19^ analysis tool is a multi-atlas label fusion technique optimized for epilepsy, developed at UCL. We used the version developed for volumetry and relaxometry^37^ which has been used in clinical studies^38,39^ and is used clinically in the NHNN.^40^

Each model was run on a Quad-Core Intel Core i5 CPU with 8Gb RAM, for comparison to clinically available hardware.

### Evaluation of metrics and statistical analysis

Dice score, Hausdorff distance, precision and recall were reported for each segmentation model. Dice score^41^ quantifies the overlap between the model-estimated segmentation and the ground truth segmentation, with a Dice score of one for a perfect segmentation, and a Dice score of zero for no overlap. The Hausdorff distance (measured in mm) quantifies the maximal distance between points located on the surface of the two segmentations. Precision and recall quantify the extent to which a model over- or under-segments respectively. A model which over-segments results in low precision, a model which under-segments results in a low recall. Statistical significance of group-level differences was assessed using the Wilcoxon signed-rank test. We adjust the significance level for multiple comparisons using the Bonferroni correction.^42^ In each dataset, we compare the metrics from InnerEye-HS to each of the four other models. Therefore, we adjusted the significance level to 0.05/4= 0.0125.

These metrics quantify the agreement between the model and ground truth segmentations. Therefore, they will negatively bias tools which use a different segmentation protocol to the ground truth segmentation. To address this bias, we consider the correlation between ground truth and segmentation volumes.

We have included a wider set of metrics and summary statistics in the Supplementary material for each of our three external test sets. This includes intersection over union (IoU), Hausdorff distance 95th percentile (HD95), average symmetric surface distance (ASSD) and volume similarity (VS), with the mean, median and 95% confidence interval reported for each metric. There are several definitions of VS.^43^ We have followed the definition used by Taha and Hanbury,^43^ which quantifies the VS as 1 minus the volume difference, where the volume difference is the absolute difference between the model and ground truth segmentation, divided by the sum of the two segmentation volumes.

## Results

### Internal validation on ADNI data

**Figure 1:**
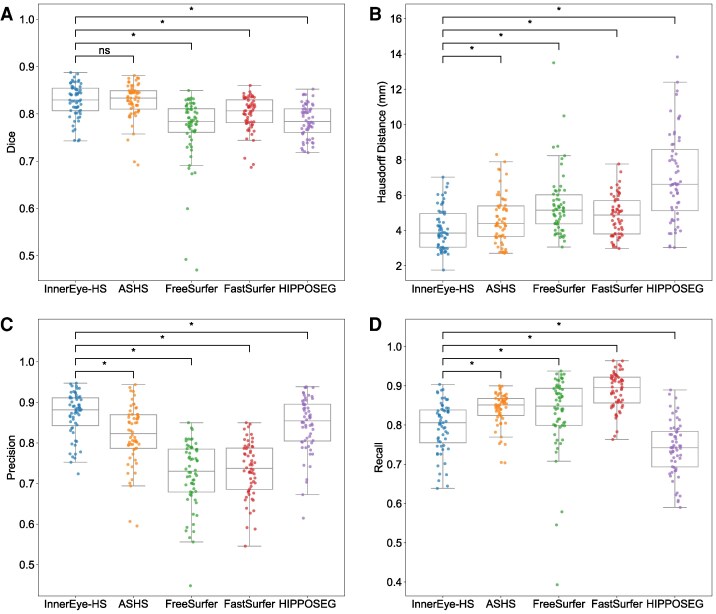
**Comparison of segmentation performance on the ADNI C dataset.** In each subplot, we plot the performance metric for each subject (*n* = 30 patients, with left and right hippocampus plotted separately) in the dataset, for each model, as a separate data point. Each subplot plots a performance metric: (**A**) Dice score, (**B**) Hausdorff distance, (**C**) precision, (**D**) recall. Data points for each model are plotted using a different colour, with the model name provided on the x-axis. The statistical significance of group differences is indicated by square brackets at the top of each graph, using the Wilcoxon signed-rank test with Bonferroni correction: * for *P*-value ≤ 0.0125, and ns for not significant, *P*-value > 0.0125. The Dice scores for InnerEye-HS segmentations were comparable to ASHS and significantly higher than FreeSurfer, FastSurfer and HIPPOSEG.

Segmentations from each model were compared to manual segmentations in the ADNI C research dataset (Fig. 1). InnerEye-HS and ASHS segmentations had the highest overlap with ground truth, demonstrated by the high Dice scores across ADNI subjects. Dice scores using InnerEye-HS were significantly higher than using FreeSurfer, FastSurfer or HIPPOSEG. FreeSurfer and FastSurfer tend to over-segment the hippocampus as demonstrated by low precision scores. HIPPOSEG tends to under-segment the hippocampus as demonstrated by the low recall scores.

### External validation on hospital data

#### NHNN dementia

**Figure 2:**
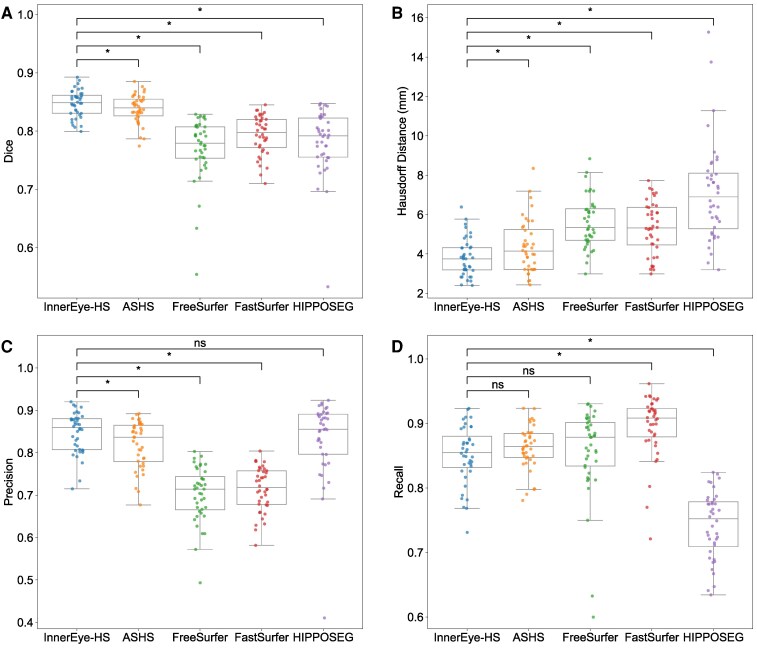
**External clinical validation of InnerEye-HS against manual segmentations in the NHNN dementia dataset.** In each subplot, we plot the performance metric for each subject (*n* = 20 patients, with left and right hippocampus plotted separately) in the dataset, for each model, as a separate data point. Each subplot plots a performance metric: (**A**) Dice score, (**B**) Hausdorff distance, (**C**) precision, (**D**) recall. Data points for each model are plotted using a different colour, with the model name provided on the x-axis. The statistical significance of group differences is indicated by square brackets at the top of each graph, using the Wilcoxon signed-rank test with Bonferroni correction: * for *P*-value ≤ 0.0125, and ns for not significant, *P*-value > 0.0125. Dice scores for InnerEye-HS segmentations were significantly higher than all other segmentation tools.

Model segmentations were further evaluated against manual segmentations in the NHNN dementia clinical dataset (Fig. 2). The Dice scores for Inner-HS segmentations are high (mean = 0.85± 0.02), demonstrating the model’s ability to generalize to external datasets. In this dataset, the Dice scores for InnerEye-HS segmentations were significantly higher (*P* ≤ 0.0125) than all other models. Consistent with the ADNI C dataset, FreeSurfer and FastSurfer tend to over-segment the hippocampus, resulting in low precision, while HIPPOSEG under-segments the hippocampus, resulting in low recall.

**Figure 3:**
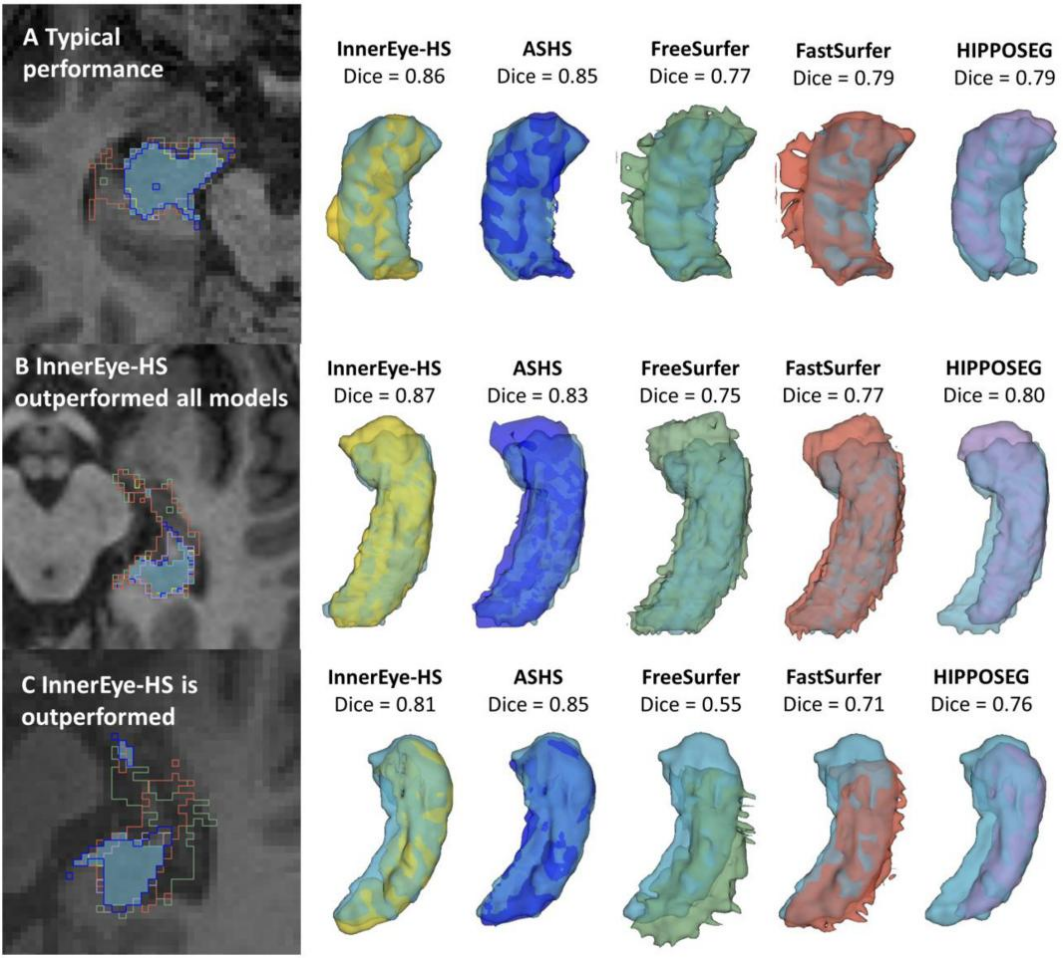
**Qualitative analysis of model performance in three scenarios from the NHNN dementia dataset.** We analyse three scenarios where: (**A**) we observe ‘typical’ performance from each of the models (Dice scores are within the middle 50th percentile for each model’s Dice scores); (**B**) InnerEye-HS outperformed all other models; and (**C**) InnerEye-HS is outperformed by another model (ASHS). Light blue segmentations represent the manual segmentations, and model segmentations are represented by: InnerEye-HS in yellow, ASHS in dark blue, FreeSurfer in green, FastSurfer in red, and HIPPOSEG in purple. InnerEye-HS and ASHS match the ground truth segmentation well. FreeSurfer and FastSurfer tend to over-segment, and HIPPOSEG tends to under-segment at the tail.

Figure 3 provides a qualitative comparison of model performance in the NHNN dementia dataset. Figure 3A illustrates a subject with ‘typical’ performance for each model. For this analysis, we randomly selected a subject where the Dice scores for each model fell within the middle 50th percentile of the model’s range of Dice scores. We observe similar performances from InnerEye-HS and ASHS, which outperform the rest of the models. FreeSurfer and FastSurfer over-segment, and HIPPOSEG under-segments, particularly at the tail. Figure 3B demonstrates a case where InnerEye-HS outperforms all other models. For this analysis, we selected the subject where the difference between the Dice scores from InnerEye-HS and the next best model was greatest. Figure 3C demonstrates a case where InnerEye-HS is most outperformed by another model. For this analysis, we selected the subject where the difference between the Dice score from InnerEye-HS and another model was greatest. While InnerEye-HS has a higher performance across all subjects, as shown in Fig. 2, in this case, InnerEye-HS is outperformed by ASHS. InnerEye-HS continues to outperform both FreeSurfer, FastSurfer and HIPPOSEG. Both FreeSurfer and FastSurfer demonstrate highly variable results compared to Fig. 3A, with areas of the head of the hippocampus missing from the segmentations, and inclusion of ventricle areas in the segmentation.

This figure also highlights differences in protocols, for example, the segmentation protocol used for HIPPSOEG does not include parts of the hippocampal tail. The tail is consistently under segmented in both cases. Supplementary Fig. 1 individually plots the contours from each model, providing a clearer visualisation of the segmentation boundaries.

#### NHNN epilepsy

**Figure 4:**
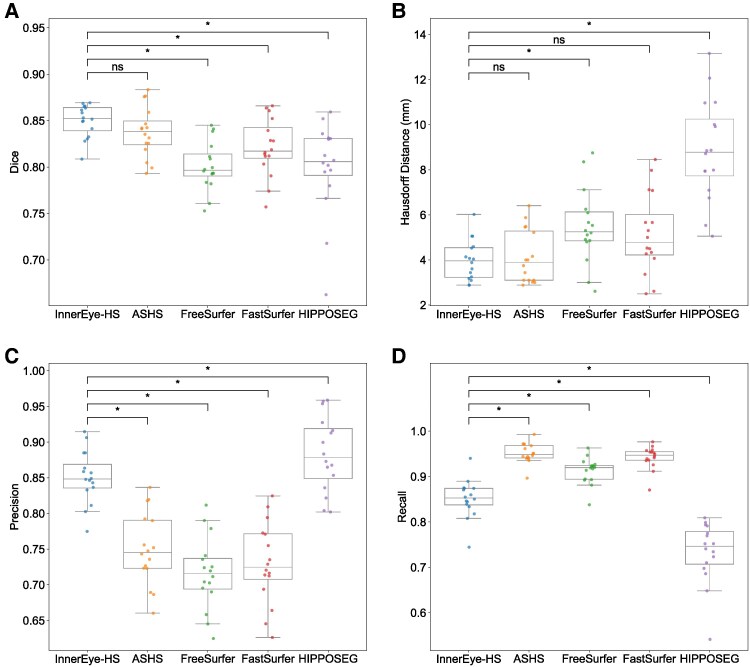
**External validation of InnerEye-HS on the NHNN epilepsy dataset.** In each subplot, we plot the performance metric for each subject (*n* = 8 patients, with left and right hippocampus plotted separately) in the dataset, for each model, as a separate data point. Each subplot plots a performance metric: (**A**) Dice score, (**B**) Hausdorff distance, (**C**) precision, (**D**) recall. Data points for each model are plotted using a different colour, with the model name provided on the x-axis . The statistical significance of group differences is indicated by square brackets at the top of each graph, using the Wilcoxon signed-rank with Bonferroni correction: * for *P*-value ≤ 0.0125, and ns for not significant, *P*-value > 0.0125. The Dice scores for InnerEye-HS segmentations were comparable to ASHS and significantly higher than FreeSurfer, FastSurfer and HIPPOSEG.

InnerEye-HS and ASHS most accurately segment the hippocampus in the NHNN epilepsy dataset, resulting in the highest average Dice score (Fig. 4). Dice scores from InnerEye-HS were significantly higher than FreeSurfer, FastSurfer and HIPPOSEG.

Specifically comparing InnerEye-HS to HIPPOSEG, which was developed for epilepsy cases and is used clinically, we observe a similar precision, but HIPPOSEG has a significantly lower recall score, meaning that its under-segments the hippocampus. As previously mentioned, the segmentation protocol used in the development of HIPPOSEG does not include parts of the tail of the hippocampus, which may explain this under-segmentation.

#### Chalfont centre for epilepsy

**Figure 5:**
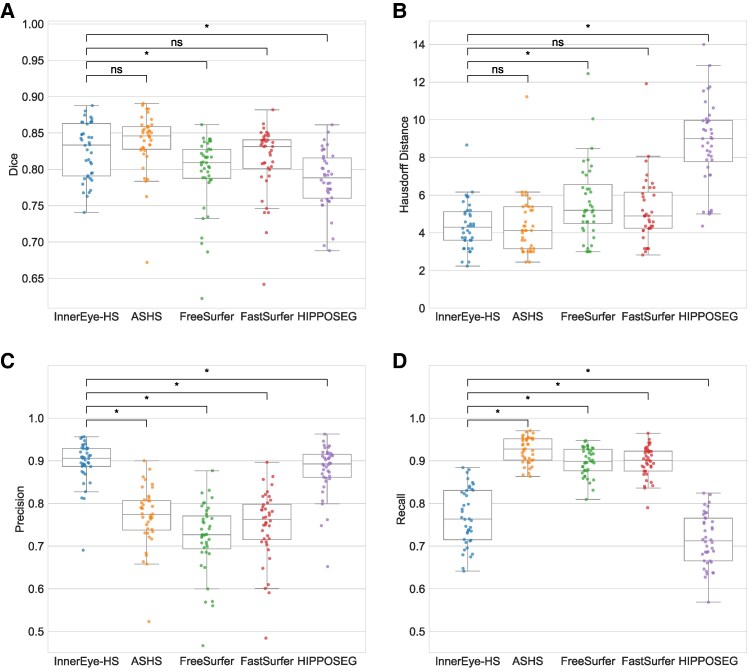
**External validation of InnerEye-HS on the Chalfont epilepsy dataset.** In each subplot, we plot the performance metric for each subject (*n* = 20, with left and right hippocampus plotted separately) in the dataset, for each model, as a separate data point. Each subplot plots a performance metric: (**A**) Dice score, (**B**) Hausdorff distance, (**C**) precision, (**D**) recall. Data points for each model are plotted using a different colour, with the model name provided on the x-axis. The statistical significance of group differences is indicated by square brackets at the top of each graph, using the Wilcoxon signed-rank with Bonferroni correction: * for *P*-value ≤ 0.0125, and ns for not significant, *P*-value > 0.0125. The Dice scores for InnerEye-HS segmentations were comparable to ASHS and FastSurfer, and significantly higher than FreeSurfer and HIPPOSEG.

InnerEye-HS, ASHS and FastSurfer most accurately segment the hippocampus in the Chalfont dataset (Fig. 5), with a non-significant difference in Dice score between InnerEye-HS and ASHS, and InnerEye-HS and FastSurfer.

#### Volumetric correlations between model and ground truth volumes

**Figure 6:**
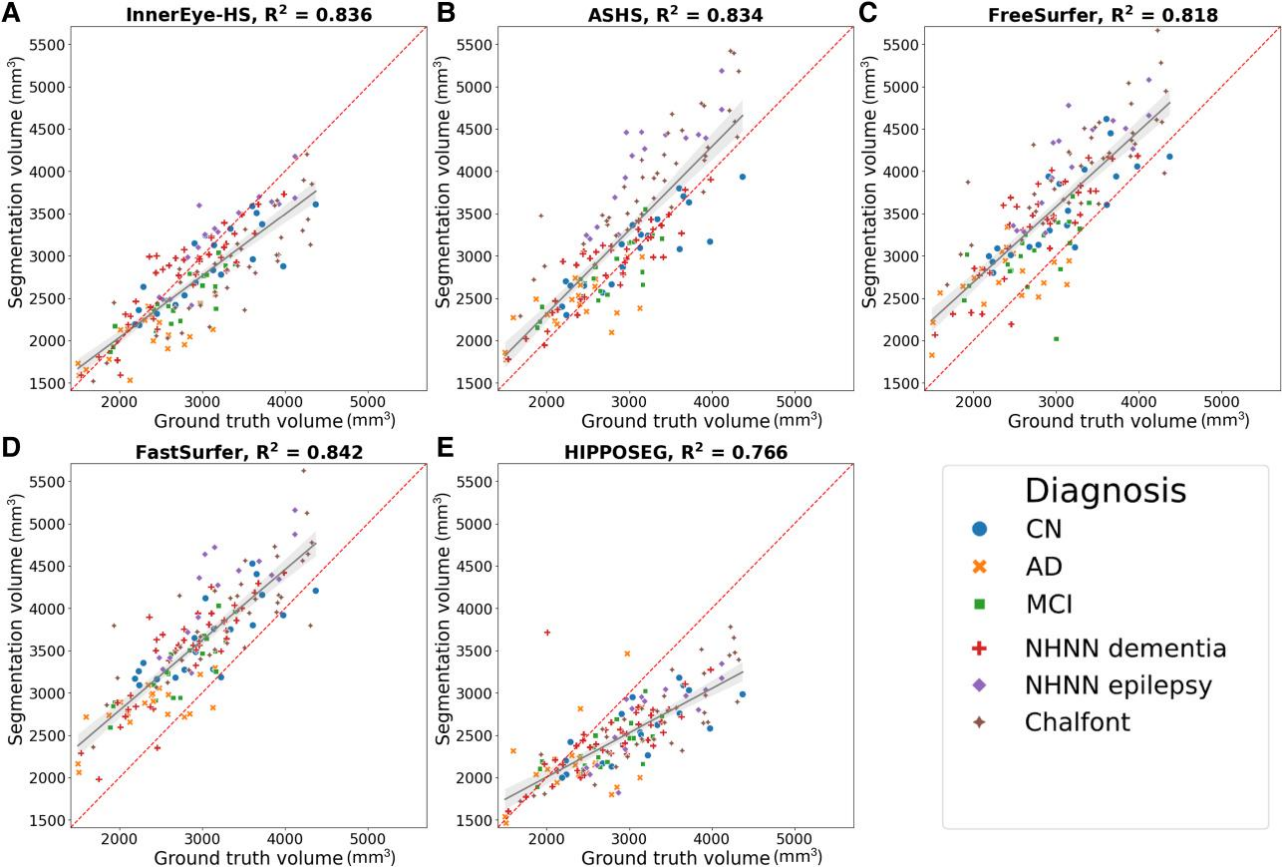
**Plots of ground truth hippocampal volume against segmentation volume for each model.** We plot results for each model on a separate subplot: (**A**) InnerEye-HS, (**B**) ASHS, (**C**) FreeSurfer, (**D**) FastSurfer, (**E**) HIPPOSEG. Each point (*n* = 156) represents a segmentation, with the colour of the plot relating to the cognitive diagnosis of the subject: cognitively normal (CN) in circles, Alzheimer's disease (Ad) in crosses, and mild cognitive impairment (MCI) in squares. No cognitive diagnoses were included in the NHNN dementia (plus symbols), NHNN epilepsy (diamonds) and Chalfont (stars) datasets. A linear regression model with the associated equation was plotted for all points, shown in a solid grey line. The identity line is shown in dashed red and Pearson's R correlation between ground truth and segmentation volumes is provided in the title of each subplot. Segmented volumes from FastSurfer are best correlated with the ground truth volumes, closely followed by InnerEye-HS.

Metrics such as Dice score, precision and recall all consider the overlap of a model segmentation and a ground truth segmentation. While this allows us to quantify the performance of each model, it is biased towards tools which are trained with the same segmentation protocol that we are testing with. Regardless of the underlying protocol, a model should be able to detect changes in overall hippocampal volume. To assess this, we plot the ground truth volume against the volume estimated by the segmentation model and calculate the variance explained by each model using Pearson’s R correlation (Fig. 6). FastSurfer is able to explain the most variance in volume (R^2^ = 0.842), followed by InnerEye-HS (R^2^ = 0.836), ASHS (R^2^ = 0.834), FreeSurfer (R^2^ = 0.818) and finally HIPPOSEG (R^2^ = 0.766). A summary of the hippocampal volumes from each model is provided in Table 2.

**Table 2:** Mean and standard deviation of segmented hippocampal volumes from the ADNI C dataset

Segmentation	Mean (std) volume in mm^2^		
	CN	MCI	Ad
Ground truth	3076.9 (609.3)	2716.5 (442.2)	2357.1 (483.6)
InnerEye-HS	2882.7 (454.5)	2468.9 (321.4)	2052.2 (280.0)
ASHS	3080.0 (463.3)	2791.6 (360.6)	2421.0 (311.4)
FreeSurfer	3549.0 (532.1)	3025.2 (391.6)	2722.5 (327.7)
FastSurfer	3635.2 (448.8)	3298.1 (375.3)	2818.6 (290.5)
HIPPOSEG	2532.5 (367.2)	2358.8 (267.3)	2171.3 (436.2)

Results are provided for each clinical diagnosis group: cognitively normal (CN), mild cognitive impairment (MCI) and Alzheimer's disease (Ad).

#### Run times

Table 3 provides mean run times for each of the segmentation tools. FastSurfer is the fastest model, followed by InnerEye-HS, ASHS, HIPPOSEG and finally FreeSurfer. FreeSurfer was the slowest model by a substantial margin, however, it is worth noting that FreeSurfer segments the whole brain, which may be beneficial to clinical applications. For InnerEye-HS, approximately a third of the runtime (4.8 min) was taken by the pre-processing steps.

**Table 3:** Average run-time for each model. Run-times were averaged across all subjects in the NHNN dementia dataset

Model	Mean (std) time in mins	Segmentation regions
InnerEye-HS	13.8 (1.1)	Hippocampus
ASHS	24.7 (1.4)	Hippocampus
FreeSurfer	299.3 (0.4)	Whole brain
FastSurfer	10.7 (0.6)	Whole brain
HIPPOSEG	57.9 (6.9)	Hippocampus

FreeSurfer and FastSurfer perform full brain segmentation, while InnerEye-HS and HIPPOSEG are hippocampal-specific. FastSurfer provides the fastest run time at 10.7 min, followed by InnerEye-HS.

## Discussion

In this work, we introduced our InnerEye hippocampal segmentation tool (InnerEye-HS) and compared it to other freely available tools on a clinical dataset for dementia and epilepsy patients. We demonstrated the accuracy of InnerEye-HS segmentations in clinical datasets, achieving high Dice scores in NHNN dementia (mean = 0.85 ± 0.02), NHNN epilepsy (mean = 0.85 ± 0.02), and Chalfont (mean = 0.83 ± 0.04) datasets. Furthermore, InnerEye-HS was found to have a high correlation between ground truth and segmentation volumes (R^2^ = 0.836), demonstrating the robustness of the segmentations to disease-related changes, and the second fastest run-time (13.8 min). This accuracy and time efficiency demonstrate the clinical advantage of InnerEye-HS over other tools.

### Comparison of segmentation methods

We compared InnerEye-HS to a selection of freely available segmentation tools, each providing different advantages and disadvantages. ASHS performed well across datasets, providing Dice scores comparable to InnerEye-HS in the ADNI (mean = 0.83 ± 0.04), NHNN epilepsy (mean = 0.84 ± 0.03) and Chalfont (mean= 0.84 ± 0.04) datasets. However, ASHS Dice scores were lower (with statistical significance) than InnerEye-HS in the NHNN dementia dataset (ASHS mean = 0.84 ± 0.03, InnerEye-HS mean= 0.85 ± 0.02), and the model had a longer run-time than InnerEye-HS (24.7 min for ASHS, 13.8 min for InnerEye-HS). Additionally, ASHS requires a different atlas for dementia and epilepsy patients, whereas one model was used across all groups for the rest of the software.

FreeSurfer and FastSurfer are established tools widely used in research which produce whole-brain segmentations, providing other clinically useful information beyond the hippocampus. However, segmentations from these models had significantly lower Dice scores across both NHNN datasets (FreeSurfer: NHNN dementia mean = 0.77 ± 0.05, NHNN epilepsy mean = 0.80 ± 0.03; FastSurfer: NHNN dementia mean = 0.79 ± 0.03, NHNN epilepsy mean = 0.82 ± 0.03), and had visible errors in the NHNN dementia dataset (Fig. 3), reducing clinical acceptability. On the Chalfont dataset, segmentations from FreeSurfer had significantly lower Dice scores than InnerEye-HS (FreeSurfer mean = 0.80± 0.05), and FastSurfer’s Dice scores were statistically comparable (FastSurfer mean = 0.81 ± 0.05). FreeSurfer had the longest run-time of all models (299.3 min), which is dramatically reduced by FastSurfer (10.7 min). It is important to note that these methods are whole-brain segmentation methods and thus have a more complicated model to fit.

HIPPOSEG was developed for epilepsy patients and is used clinically at the NHNN. The tool produces a clinical report providing context for the segmentations, e.g. volumetric comparisons to a normative population. However, the segmentations were found to have relatively low Dice scores across all clinical datasets (NHNN dementia mean = 0.78 ± 0.06, NHNN epilepsy mean= 0.80 ± 0.05, Chalfont mean = 0.79 ± 0.04), long run-time (57.9 min), and the lowest correlation between segmented and ground truth volumes (R^2^ = 0.71).

### Ground truth segmentation

Manual segmentation of medical images is known to suffer from high inter-rater variability.^44,45^ Boundaries of hippocampal regions will vary between different protocols and between the individual raters’ outlines.^31,46^ This variability affects both the training and evaluation of downstream segmentation tools.

In this work, we pre-trained our InnerEye-HS models on a large dataset of MRI scans with semi-automatic segmentations (*N* = 1250)^28^ and fine-tuned the model on a smaller dataset of manual segmentations from the HarP dataset (*N* = 135),^31^ thus exposing our model to a larger number of MRI scans, while leveraging the accuracy of the HarP dataset. This dataset has been segmented by five expert raters according to a consensus-agreed protocol for hippocampal segmentation.^31^ This allowed us to minimize errors from the semi-automatic segmentation method, however, the model will still be affected by some inter-rater variability in the HarP training segmentations.

In our evaluation datasets (ADNI C, NHNN dementia, NHNN epilepsy and Chalfont), manual segmentations were traced by a local clinician and confirmed by a consultant neuroradiologist, which we took to be ground truth. This is likely to increase errors in the segmentations, due to a smaller number of raters compared to the HarP dataset. Furthermore, evaluating the Dice score against this ground truth will negatively bias segmentation tools which have been trained on a different protocol. In this work, HIPPOSEG will be negatively biased by our Dice score evaluation, as the tail of the hippocampus is excluded from the protocol used for HIPPOSEG and is included in the protocol for the ground truth segmentations used to evaluate all models. Similarly, the exclusion of fimbria from our manual clinical protocol may have a small negative impact on Dice scores of tools that include the fimbria in the training. This would also impact InnerEye-HS.

### Steps to clinical implementation

Despite the advantages of InnerEye-HS, there are opportunities for further work to aid clinical utility. Firstly, the model has only been validated on data collected using the MPRAGE protocol, the same protocol used to collect the training data. We have not demonstrated the generalisability of InnerEye-HS to unseen imaging protocols (i.e. non-MPRAGE imaging protocols). This deviation from the training dataset is a typical point of failure for deep learning models and can result in a reduction in accuracy.^15,47,48^ Furthermore, MRI scans from the NHNN and epilepsy datasets were pre-processed prior to InnerEye-HS inference, such that resolution and cropping were consistent with the training dataset. These pre-processing steps ensure good segmentations from InnerEye-HS at the cost of increased running time. Models such as Synthseg^15^ have shown great success in improving the generalisability of segmentation models through data augmentation.^49,50^ Data augmentation could remove the need for pre-processing steps while increasing the model’s robustness in handling different imaging protocols. Future work will use data augmentation to incorporate synthetic data into the training process and carry out validation on different imaging protocols.

Integration of InnerEye-HS into clinical pipelines will further enable clinical uptake.^51^ We have produced a Docker version of InnerEye-HS to ensure the model is straightforward to install and run on a standard CPU. Future work will incorporate InnerEye-HS segmentations into quantitative reports, similar to those developed by Vos *et al*.,^37^ providing visualisation of the segmentations alongside volume calculations and comparisons to datasets of healthy subjects. This will require close collaboration with clinicians to ensure suitability for end-users and to carry out a clinical validation study of the quantitative reports. Further work will also incorporate an uncertainty metric to predict poor segmentations for review,^52,53^ improving reliability in the model outputs.

The metrics we have presented here provide important markers, however, there are further considerations which will be vital for clinical utility. Factors include: the ease of incorporating software into clinical systems; whether computing resource requirements are suitable for a clinical setting; the requirement of cloud resources and the resultant implications on patient data security; the extent of internal technical support available, and the software’s robustness to bugs. These factors will be critical when reviewing which software to implement in a clinical environment. Furthermore, while we have shown that segmentations using InnerEye-HS can produce high Dice scores in our clinical datasets, we have not established whether these Dice scores are high enough to be suitable for clinical applications. Future work will evaluate this through additional clinical validation, where clinicians will assess quantitative reports generated using InnerEye-HS on a patient-by-patient basis.

## Conclusion

In conclusion, we have presented InnerEye-HS, a new hippocampal segmentation tool, and demonstrated its accuracy in clinical datasets and robustness to disease-related changes. Future work will aim to improve the generalisability of the model and incorporate the model into reports to enable smooth uptake in clinical settings.

## Supplementary Material

fcag183_Supplementary_Data

## Data Availability

The ADNI is a public dataset available to download at www.adni-info.org. The NHNN dementia and epilepsy datasets are not available for wider use due to ethical limitations on data sharing.
