# Supplementary material for: InnerEye-HS: a disease-agnostic clinical tool for hippocampal segmentation
Source: Brain Commun. 2026 May 21;8(3):fcag183. doi: 10.1093/braincomms/fcag183 (PMC13245398; doi:10.1093/braincomms/fcag183)
Supplement: fcag183_Supplementary_Data [file fcag183_supplementary_data.docx]

**
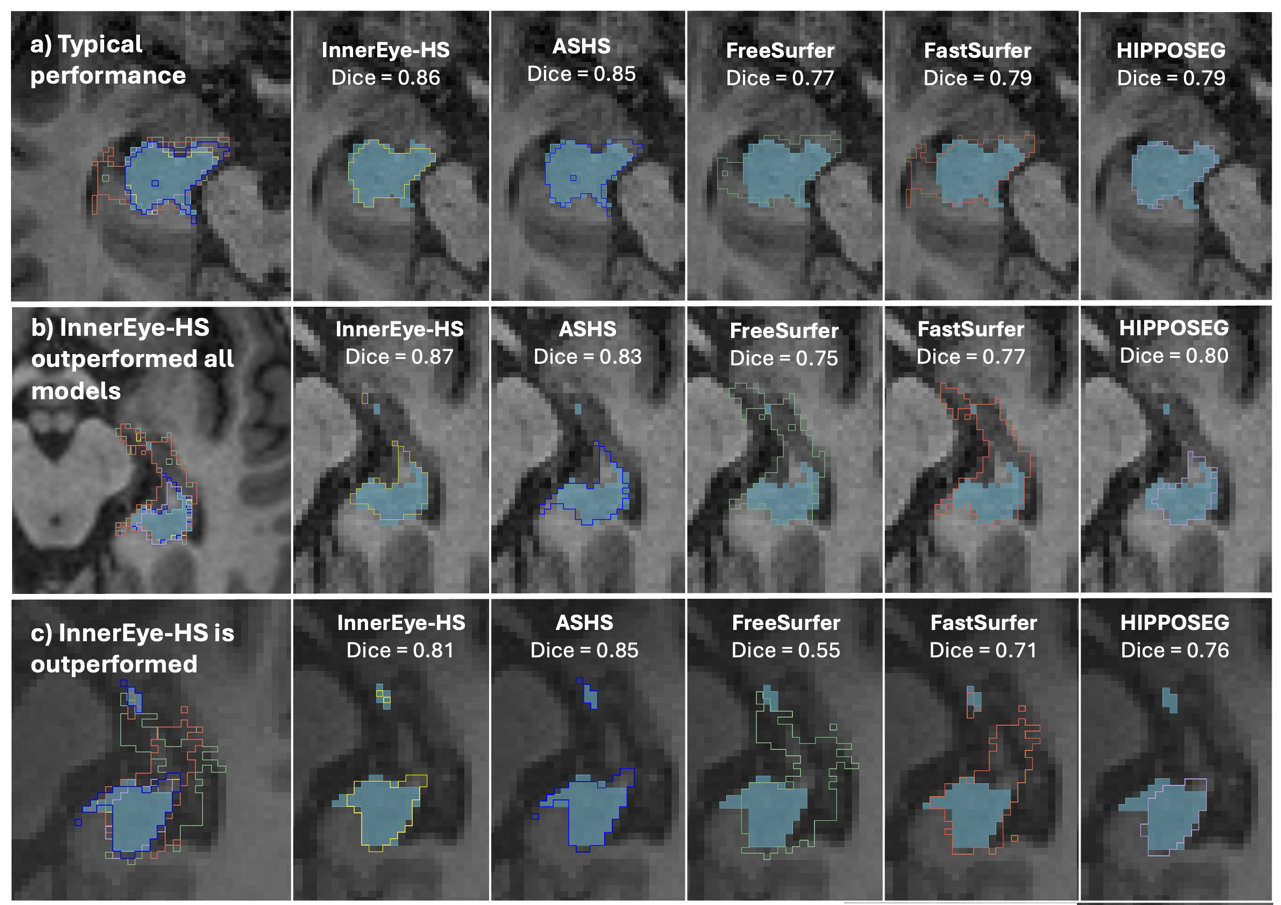
**

**Supplementary Figure 1: A qualitative comparison of segmentation contours using each segmentation tool, demonstrated in three scenarios from the NHNN dementia dataset. Light blue segmentations represent the manual segmentations, and model segmentations are represented by: InnerEye-HS in yellow, ASHS in dark blue, FreeSurfer in green, FastSurfer in red, and HIPPOSEG in purple. This figure provides an extension of the results displayed in Figure 3 in the main text.**

**Supplementary Table 1: Extended metrics for the NHNN dementia dataset**

| **Metric** |  | **Model** | | | | |
| --- | --- | --- | --- | --- | --- | --- |
|  |  | **InnerEye-HS** | **ASHS** | **FreeSurfer** | **FastSurfer** | **HIPPOSEG** |
| Dice | Mean | **0.85** | 0.84 | 0.77 | 0.79 | 0.78 |
|  | Median | **0.85** | 0.84 | 0.78 | 0.80 | 0.79 |
|  | 95% | **0.88** | 0.87 | 0.83 | 0.84 | 0.84 |
| IoU | Mean | **0.74** | 0.72 | 0.63 | 0.66 | 0.64 |
|  | Median | **0.74** | 0.72 | 0.64 | 0.66 | 0.66 |
|  | 95% | **0.79** | 0.77 | 0.70 | 0.72 | 0.73 |
| Precision | Mean | **0.85** | 0.82 | 0.70 | 0.71 | 0.83 |
|  | Median | **0.86** | 0.84 | 0.71 | 0.72 | **0.86** |
|  | 95% | **0.91** | 0.88 | 0.79 | 0.78 | **0.91** |
| Recall | Mean | 0.85 | 0.86 | 0.86 | **0.90** | 0.74 |
|  | Median | 0.85 | 0.86 | 0.88 | **0.91** | 0.75 |
|  | 95% | 0.91 | 0.91 | 0.93 | **0.94** | 0.82 |
| HD | Mean | **3.81** | 4.39 | 5.60 | 5.33 | 7.11 |
|  | Median | **3.75** | 4.14 | 5.34 | 5.32 | 6.90 |
|  | 95% | **5.52** | 6.88 | 7.95 | 7.28 | 11.40 |
| HD95 | Mean | **1.59** | 1.74 | 2.55 | 2.38 | 2.80 |
|  | Median | **1.49** | 1.60 | 2.40 | 2.40 | 2.62 |
|  | 95% | **1.94** | 2.71 | 4.51 | 3.34 | 4.39 |
| ASSD | Mean | **0.53** | 0.56 | 0.82 | 0.75 | 0.87 |
|  | Median | **0.53** | 0.55 | 0.78 | 0.75 | 0.83 |
|  | 95% | **0.61** | 0.70 | 1.24 | 0.96 | 1.20 |
| VS | Mean | **0.96** | **0.96** | 0.90 | 0.89 | 0.92 |
|  | Median | **0.97** | **0.97** | 0.89 | 0.89 | 0.92 |
|  | 95% | **1.00** | **1.00** | 0.97 | 0.95 | 0.99 |

We provide the mean, median and 95% confidence interval for the following metrics: Dice score, intersection over union (IoU), precision, recall, Hausdorff distance (HD), Hausdorff distance 95^th^ percentile (HD95), average symmetric surface distance (ASSD) and volume similarity (VS). The best model’s result for each row is highlighted in bold. With the exception of recall, InnerEye-HS consistently provides the best, or joint best results for each metric.

**Supplementary Table 2: Extended metrics for the NHNN epilepsy dataset**

| **Metric** |  | **Model** | | | | |
| --- | --- | --- | --- | --- | --- | --- |
|  |  | **InnerEye-HS** | **ASHS** | **FreeSurfer** | **FastSurfer** | **HIPPOSEG** |
| Dice | Mean | **0.85** | 0.84 | 0.80 | 0.82 | 0.80 |
|  | Median | **0.85** | 0.84 | 0.80 | 0.82 | 0.81 |
|  | 95% | 0.87 | **0.88** | 0.84 | 0.86 | 0.85 |
| IoU | Mean | **0.74** | 0.72 | 0.67 | 0.70 | 0.67 |
|  | Median | **0.74** | 0.72 | 0.66 | 0.69 | 0.67 |
|  | 95% | 0.77 | **0.78** | 0.73 | 0.76 | 0.74 |
| Precision | Mean | 0.85 | 0.75 | 0.72 | 0.73 | **0.88** |
|  | Median | 0.85 | 0.75 | 0.72 | 0.72 | **0.88** |
|  | 95% | 0.91 | 0.82 | 0.80 | 0.81 | **0.96** |
| Recall | Mean | 0.85 | **0.95** | 0.91 | 0.94 | 0.73 |
|  | Median | 0.85 | **0.95** | 0.92 | **0.95** | 0.75 |
|  | 95% | 0.90 | **0.98** | 0.95 | 0.97 | 0.80 |
| HD | Mean | **3.98** | 4.12 | 5.47 | 5.15 | 8.87 |
|  | Median | 3.96 | **3.88** | 5.25 | 4.76 | 8.78 |
|  | 95% | **5.30** | 6.01 | 8.45 | 8.10 | 12.34 |
| HD95 | Mean | **1.52** | 1.84 | 2.13 | 2.09 | 3.19 |
|  | Median | **1.50** | 1.68 | 1.98 | 1.95 | 3.14 |
|  | 95% | **1.84** | 2.58 | 3.37 | 3.29 | 4.43 |
| ASSD | Mean | **0.52** | 0.61 | 0.70 | 0.66 | 0.85 |
|  | Median | **0.51** | 0.59 | 0.66 | 0.62 | 0.83 |
|  | 95% | **0.60** | 0.81 | 0.95 | 0.91 | 1.18 |
| VS | Mean | **0.97** | 0.88 | 0.88 | 0.87 | 0.91 |
|  | Median | **0.97** | 0.88 | 0.88 | 0.87 | 0.90 |
|  | 95% | **1.00** | 0.94 | 0.94 | 0.94 | 0.97 |

We provide the mean, median and 95% confidence interval for the following metrics: Dice score, intersection over union (IoU), precision, recall, Hausdorff distance (HD), Hausdorff distance 95^th^ percentile (HD95), average symmetric surface distance (ASSD) and volume similarity (VS). The best model’s result for each row is highlighted in bold. In general, InnerEye-HS and ASHS provide the best performance except for precision, where HIPPOSEG outperforms all other models.

**Supplementary Table 3: Extended metrics for the Chalfont dataset**

| **Metric** |  | **Model** | | | | |
| --- | --- | --- | --- | --- | --- | --- |
|  |  | **InnerEye-HS** | **ASHS** | **FreeSurfer** | **FastSurfer** | **HIPPOSEG** |
| Dice | Mean | 0.83 | **0.84** | 0.80 | 0.81 | 0.79 |
|  | Median | 0.83 | **0.85** | 0.81 | 0.83 | 0.79 |
|  | 95% | 0.87 | **0.88** | 0.84 | 0.86 | 0.84 |
| IoU | Mean | 0.70 | **0.72** | 0.66 | 0.69 | 0.65 |
|  | Median | 0.71 | **0.73** | 0.68 | 0.71 | 0.65 |
|  | 95% | 0.78 | **0.79** | 0.73 | 0.75 | 0.73 |
| Precision | Mean | **0.90** | 0.77 | 0.72 | 0.75 | 0.88 |
|  | Median | **0.91** | 0.77 | 0.73 | 0.76 | 0.89 |
|  | 95% | **0.95** | 0.86 | 0.83 | 0.86 | 0.94 |
| Recall | Mean | 0.77 | **0.92** | 0.90 | 0.90 | 0.72 |
|  | Median | 0.76 | **0.93** | 0.90 | 0.90 | 0.71 |
|  | 95% | 0.87 | **0.97** | 0.94 | 0.94 | 0.80 |
| HD | Mean | **5.16** | 5.18 | 6.31 | 5.91 | 9.46 |
|  | Median | 4.30 | **4.12** | 5.20 | 4.90 | 9.00 |
|  | 95% | **6.29** | 6.42 | 10.17 | 8.25 | 12.94 |
| HD95 | Mean | **1.72** | 1.90 | 2.36 | 2.18 | 3.36 |
|  | Median | **1.46** | 1.73 | 2.24 | 2.00 | 3.31 |
|  | 95% | **2.24** | 2.47 | 3.80 | 3.52 | 5.10 |
| ASSD | Mean | **0.61** | 0.62 | 0.76 | 0.70 | 0.91 |
|  | Median | **0.58** | 0.59 | 0.71 | 0.64 | 0.93 |
|  | 95% | **0.76** | 0.78 | 1.14 | 0.97 | 1.20 |
| VS | Mean | **0.92** | 0.91 | 0.89 | 0.90 | 0.89 |
|  | Median | **0.91** | **0.91** | 0.89 | **0.91** | 0.89 |
|  | 95% | **0.99** | 0.98 | 0.97 | 0.98 | 0.98 |

We provide the mean, median and 95% confidence interval for the following metrics: Dice score, intersection over union (IoU), precision, recall, Hausdorff distance (HD), Hausdorff distance 95^th^ percentile (HD95), average symmetric surface distance (ASSD) and volume similarity (VS). The best model’s result in each row is highlighted in bold. InnerEye-HS and ASHS provide the best performance over all metrics, with FastSurfer performing equally well for median VS.
